# Impact of hematologic malignancy and type of cancer therapy on COVID-19 severity and mortality: lessons from a large population-based registry study

**DOI:** 10.1186/s13045-020-00970-7

**Published:** 2020-10-08

**Authors:** Julio García-Suárez, Javier de la Cruz, Ángel Cedillo, Pilar Llamas, Rafael Duarte, Víctor Jiménez-Yuste, José Ángel Hernández-Rivas, Rodrigo Gil-Manso, Mi Kwon, Pedro Sánchez-Godoy, Pilar Martínez-Barranco, Blanca Colás-Lahuerta, Pilar Herrera, Laurentino Benito-Parra, Adrián Alegre, Alberto Velasco, Arturo Matilla, María Concepción Aláez-Usón, Rafael Martos-Martínez, Carmen Martínez-Chamorro, Keina Susana-Quiroz, Juan Francisco Del Campo, Adolfo de la Fuente, Regina Herráez, Adriana Pascual, Elvira Gómez, Jaime Pérez-Oteyza, Elena Ruiz, Arancha Alonso, José González-Medina, Lucía Núñez Martín-Buitrago, Miguel Canales, Isabel González-Gascón, María Carmen Vicente-Ayuso, Susana Valenciano, María García Roa, Pablo Estival Monteliu, Javier López-Jiménez, Cristián Escolano Escobar, Javier Ortiz-Martín, José Luis Diez-Martin, Joaquín Martinez-Lopez, the Asociación Madrileña de Hematología y Hemoterapia (AMHH), Cristina Serí-Merino, Keina Queiroz-Cervantes, Mónica Estévez Fernandez, María-José Peñalva-Moreno, Daniel Naya-Errea, Laura Bermejo-Martínez, Laura Llorente-González

**Affiliations:** 1 Hematology Department, University Hospital Príncipe de Asturias, Alcalá de Henares, Madrid, Spain; 2 Research Institute imas12, University Hospital, SAMID-ISCIII, 12 de Octubre, Madrid, Spain; 3 Asociación Madrileña de Hematología Y Hemoterapia (AMHH), Madrid, Spain; 4 Hematology Department, University Hospital Fundación Jiménez Díaz, Madrid, Spain; 5 Hematology Department, University Hospital Puerta de Hierro Majadahonda, Madrid, Spain; 6 Hematology Department, University Hospital La Paz, Madrid, Spain; 7 Hematology Department, University Hospital Infanta Leonor, Madrid, Spain; 8 Hematology Department, CNIO-ISCIII, CIBERONC, Complutense University, Hospital 12 de Octubre, imas12 Madrid, Spain; 9 Hematology Department, University General Hospital Gregorio Marañón, Instituto de Investigación Sanitaria Gregorio Marañón, Madrid, Spain; 10 Department of Medicine, Complutense University, Madrid, Spain; 11 Hematology Department, University Hospital Severo Ochoa, Madrid, Spain; 12 Hematology Department, University Hospital Fundación Alcorcón, Madrid, Spain; 13 Hematology Department, University Hospital Clínico San Carlos, Madrid, Spain; 14 Hematology Department, University Hospital Ramón Y Cajal, Madrid, Spain; 15 Hematology Department, University Hospital Getafe, Madrid, Spain; 16 Hematology Department, University Hospital La Princesa, Madrid, Spain; 17 Hematology Department, University Hospital Rey Juan Carlos, Móstoles, Madrid, Spain; 18 Hematology Department, University Hospital Central de La Defensa Gómez Ulla, Madrid, Spain; 19 Hematology Department, University Hospital HLA Moncloa, Madrid, Spain; 20 Hematology Department, University Hospital Villalba, Villalba, Madrid, Spain; 21 Hematology Department, University Hospital Quirónsalud, Pozuelo de Alarcón, Madrid, Spain; 22 Hematology Department, University Hospital Móstoles, Madrid, Spain; 23 Hematology Department, University Hospital Henares, Coslada, Madrid, Spain; 24 Hematology Department, MD Anderson Cancer Center Madrid, Madrid, Spain; 25 Hematology Department, University Hospital Infanta Sofía, San Sebastián de Los Reyes, Madrid, Spain; 26 Hematology Department, University Hospital Infanta Elena, Valdemoro, Madrid, Spain; 27 Hematology Department, University Hospital Sureste, Arganda del Rey, Madrid, Spain; 28 Hematology Department, University Hospital HM Sanchinarro, Madrid, Spain; 29 Hematology Department, University Hospital Tajo, Aranjuez, Madrid, Spain; 30 Hematology Department, Hospital Ruber, Madrid, Spain; 31 i+12, CNIO-ISCIII, Hospital 12 de Octubre, Servicio de Hematología, Centro de Actividades Ambulatorias, Planta Tercera Bloque D, Univ. Complutense, Avd de Cordoba s/n, 28041 Madrid, Spain

**Keywords:** Severe acute respiratory syndrome coronavirus 2 (SARS-CoV-2), COVID-19, Hematologic neoplasms

## Abstract

**Background:**

Patients with cancer have been shown to have a higher risk of clinical severity and mortality compared to non-cancer patients with COVID-19. Patients with hematologic malignancies typically are known to have higher levels of immunosuppression and may develop more severe respiratory viral infections than patients with solid tumors. Data on COVID-19 in patients with hematologic malignancies are limited. Here we characterize disease severity and mortality and evaluate potential prognostic factors for mortality.

**Methods:**

In this population-based registry study, we collected de-identified data on clinical characteristics, treatment and outcomes in adult patients with hematologic malignancies and confirmed severe acute respiratory syndrome coronavirus-2 (SARS-CoV-2) infection within the Madrid region of Spain. Our case series included all patients admitted to 22 regional health service hospitals and 5 private healthcare centers between February 28 and May 25, 2020. The primary study outcome was all-cause mortality. We assessed the association between mortality and potential prognostic factors using Cox regression analyses adjusted for age, sex, comorbidities, hematologic malignancy and recent active cancer therapy.

**Results:**

Of 833 patients reported, 697 were included in the analyses. Median age was 72 years (IQR 60–79), 413 (60%) patients were male and 479 (69%) and 218 (31%) had lymphoid and myeloid malignancies, respectively. Clinical severity of COVID-19 was severe/critical in 429 (62%) patients. At data cutoff, 230 (33%) patients had died. Age ≥ 60 years (hazard ratios 3.17–10.1 vs < 50 years), > 2 comorbidities (1.41 vs ≤ 2), acute myeloid leukemia (2.22 vs non-Hodgkin lymphoma) and active antineoplastic treatment with monoclonal antibodies (2·02) were associated with increased mortality; conventional chemotherapy showed borderline significance (1.50 vs no active therapy). Conversely, Ph-negative myeloproliferative neoplasms (0.33) and active treatment with hypomethylating agents (0.47) were associated with lower mortality. Overall, 574 (82%) patients received antiviral therapy. Mortality with severe/critical COVID-19 was higher with no therapy vs any antiviral combination therapy (2.20).

**Conclusions:**

In this series of patients with hematologic malignancies and COVID-19, mortality was associated with higher age, more comorbidities, type of hematological malignancy and type of antineoplastic therapy. Further studies and long-term follow-up are required to validate these criteria for risk stratification.

## Background

Severe acute respiratory syndrome coronavirus 2 (SARS-CoV-2) and coronavirus disease 2019 (COVID-19) [1] have resulted in a World Health Organization (WHO)-classified pandemic [2]. Most patients with SARS-CoV-2 infection are asymptomatic or exhibit mild-to-moderate symptoms, but approximately 15% progress to severe pneumonia, and 5% require intensive care unit (ICU) management due to acute respiratory distress syndrome, septic shock and/or multiple organ failure. As of July 29, 2020, 16,708,920 cases of COVID-19 have been reported, including 660,123 deaths [3]. Case fatality is reported at 3.95% [3], but this varies widely by location [3].

Poor-risk factors for outcome in COVID-19 patients include old age, hypertension, cardiovascular disease and diabetes [4]. Cancer patients also appear to have a worse prognosis [5]. A meta-analysis found that cancer prevalence in people with COVID-19 was 2% [6]. More importantly, patients with cancer had a higher risk of severe events (admission to an ICU requiring invasive ventilation, or death) compared to those without cancer (11–39% vs 5.8–7.6%) [7, 8]. A large-scale study using UK Coronavirus Cancer Monitoring Project data gave consistent findings [9].

Patients with hematologic malignancies usually have higher levels of immunosuppression and may develop more severe respiratory viral infections than patients with solid tumors [10]. In Europe and the USA, hematologic malignancies comprise the fourth most common cancer site [11, 12]. The use of new antineoplastic agents, particularly novel targeted therapies, has improved overall survival. However, these therapies have side effects on humoral and cell-mediated immunity, increasing the risk of infections caused by viral agents [13]. To date, few data are available on COVID-19 in patients with hematologic malignancies. Reported studies have focused on hospitalized patients. One showed very high mortality (40% at 1 month) among 25 patients in France [14]. A second suggested that hospitalized patients with hematologic malignancies have a higher mortality rate than patients without hematologic malignancies (62% vs 8%) [15]. However, these were small patient series; the clinical impact of COVID-19 in this population remains unclear. Therefore, real-time collection, analysis, and dissemination of data about COVID-19 in patients with hematologic malignancies and their outcomes are needed. Meanwhile, impactful decisions are being suggested on the basis of expert opinion [16, 17].

The Madrid region was the epicenter of Spain’s COVID-19 crisis. The first cases were declared in Spain on January 31 and in the Madrid region on February 25, 2020. As of 29 July, 78,807 patients had been diagnosed with COVID‐19 and 15,199 fatalities reported in the Madrid region [18]. We considered it critical to collect clinical data in patients with hematologic malignancies within a defined geographical area with high excess mortality in order to understand the epidemiology of COVID-19. We aimed to identify independent prognostic factors for mortality that could support recommendations for managing patients with hematologic malignancies in healthcare emergency situations such as the COVID-19 pandemic.

## Methods

### Study design and participants

This was a multicenter, registry-based study with prospective data collection sponsored by the Madrid Society of Hematology (Asociación Madrileña de Hematología y Hemoterapia, AMHH). AHMM established the registry on March 13 by contacting all members to register patients with hematologic malignancies who had microbiological confirmation of SARS-CoV-2 infection. Health care for patients with hematologic malignancies in the Madrid region is provided at 26 hospitals affiliated with the Madrid regional health service (Servicio Madrileño de Salud, SERMAS), covering a population of 6.6 million inhabitants. Additionally, patients are seen at six private non-SERMAS-affiliated healthcare centers. This case series included consecutive patients with hematologic malignancies aged ≥ 18 years who received a confirmed diagnosis of COVID-19 in the emergency departments, hospital wards (patients infected while hospitalized) or outpatient clinics of these Madrid hospitals up to May 25, 2020. Clinical specimens for diagnosis confirmation were obtained by nasopharyngeal swab collection in accordance with Spanish disease control and prevention guidelines. Samples were processed at local microbiology laboratories, and SARS-CoV-2 one-step real-time reverse transcriptase PCR diagnostic assay was performed [19].

The study was approved by the Institutional Review Board (IRB) of University Hospital 12 de Octubre (n 20/189) and then by the IRBs of all participating centers. Written informed consent was waived in light of the urgent need to collect and report data. The study was performed in accordance with the principles of the Declaration of Helsinki and the International Conference on Harmonization Good Clinical Practice guidelines.

### Procedures

Data were prospectively extracted locally from electronic health records by hospital hematology department study coordinators and uploaded to a secure web platform (HEMATO-MADRID COVID-19), which utilized REDCap data capture tools and was supported by AMHH. Anonymized data were centrally processed by a coordinating team at AMHH and checked for duplicates. Clinical management decisions were made according to local protocols at each center; national guidelines for COVID-19 treatment issued by the Spanish Ministry of Health, National Health System, and National Medicine Agency were widely implemented. Decisions about hospital/ICU admissions were made locally based on daily updated criteria during the healthcare emergency period.

Potential prognostic factors were collected including pre-infection patient characteristics (age, sex, comorbidities, type of hematologic malignancy and therapy), COVID-19 clinical severity, treatments and care setting. ‘Active antineoplastic treatment’ was defined as having received anticancer therapy within 30 days prior to COVID-19 diagnosis. Therapies were classified as ‘conventional chemotherapy,’ ‘low-intensity chemotherapy,’ hypomethylating agents, monoclonal antibodies, immunomodulatory drugs, ‘molecular targeted therapies,’ or supportive care (Table 1). Patients who were receiving monoclonal antibodies in combination with cytotoxic chemotherapy were classified into the category of ‘conventional chemotherapy.’ COVID-19 severity classification followed WHO guidelines [20]. No data on symptoms, laboratory findings, respiratory support, or viral kinetics were available for analyses as standardization was not achieved.

**Table 1:** Characteristics and outcomes of patients with hematologic malignancies and COVID-19, according to the type of malignancy

		Lymphoid malignancies					Myeloid malignancies			
	Total	NHL	MM	CLL	HL	ALL	MDS	AML	CML	Ph-MPN
*Patients*	697 (100)	187 (27)	137 (20)	109 (15)	33 (5)	13 (2)	78 (11)	61 (9)	16 (2)	63 (9)
Age, years	72 (60–79)	70 (58–77)	73 (65–79)	73 (60–81)	59 (45–75)	45 (37–55)	80 (71–86)	65 (52–76)	61 (48–74)	75 (66–83)
Sex*	% shown as proportion of all patients with that malignancy									
Female	277 (40)	81 (44)	56 (41)	39 (36)	12 (38)	7 (54)	27 (35)	32 (53)	3 (20)	20 (32)
Male	413 (60)	104 (56)	81 (59)	68 (64)	20 (62)	6 (46)	51 (65)	28 (47)	12 (80)	43 (68)
*Comorbidities, n*	2 (1–3)	1 (1–2)	2 (1–3)	1 (1–3)	1 (1–3)	1 (0–1)	2 (1–3)	1 (0–2)	1 (0–3)	2 (1–3)
Cardiac disease	138 (20)	33 (18)	30 (22)	20 (18)	8 (24)	1 (8)	22 (28)	7 (11)	3 (13)	14 (22)
Pulmonary disease	90 (13)	21 (11)	20 (15)	16 (15)	2 (6)	1 (8)	14 (18)	6 (10)	4 (25)	6 (10)
Renal disease	77 (11)	11 (6)	32 (23)	8 (7)	4 (12)	1 (8)	9 (12)	22 (36)	4 (25)	9 (14)
Hypertension	277 (40)	64 (34)	63 (46)	44 (40)	13 (39)	2 (15)	35 (45)	1 (2)	2 (13)	30 (48)
Diabetes	121 (17)	29 (16)	29 (21)	20 (18)	7 (21)	0	18 (23)	5 (8)	3 (19)	10 (16)
Other cancer	79 (11)	19 (10)	14 (10)	13 (12)	1 (3)	2 (15)	16 (21)	2 (3)	1 (6)	11 (17)
*Active antineoplastic treatment*^*†*^	% shown as proportion of all patients with that malignancy									
Conventional chemotherapy^‡^	127 (18)	54(29)	29 (21)	2 (2)	9 (28)	6 (50)	1 (1)	26 (42)	0	0
Low-intensity chemotherapy^§^	42 (6)	1 (1)	4 (3)	2 (2)	0	1 (8)	1 (1)	0	2 (12)	31 (49)
Molecular targeted therapies^¶^	82 (12)	7 (4)	12 (9)	28 (26)	0	0	0	3 (5)	14 (88)	18 (29)
Monoclonal antibodies^‡‡^	44 (6)	25 (13)	14 (10)	1 (1)	4 (13)	0	0	0	0	0
Immunomodulatory drugs^ǁ^	45 (7)	0	45 (33)	0	0	0	0	0	0	0
Hypomethylating agents**	33 (5)	1 (1)	0	0	0	0	15 (19)	17 (28)	0	0
Supportive care^††^	12 (2)	2 (1)	0	0	0	0	5 (7)	0	0	5 (8)
Not detailed	24 (3)	5 (2)	6 (4)	1 (1)	5 (16)	1 (8)	2 (3)	0	0	4 (6)
No active therapy	286 (41)	92 (49)	27 (20)	75 (69)	14 (44)	4 (33)	54 (69)	15 (25)	0	5 (8)
*Care setting*	% shown as proportion of all patients with that malignancy									
Ambulatory	89 (13)	24 (13)	16 (12)	15 (14)	4 (12)	3 (23)	6 (8)	9 (15)	6 (37)	9 (14)
Hospital	608 (87)	163 (87)	121 (88)	97 (89)	29 (88)	10 (77)	72 (92)	52 (85)	10 (63)	54 (86)
*Outcome*										
Survivors	467 (67)	128 (68)	90 (66)	70 (64)	24 (73)	11 (85)	45 (58)	34 (56)	14 (87)	51 (81)
Non-survivors	230 (33)	59 (32)	47 (34)	39 (36)	9 (27)	2 (15)	33 (42)	27 (44)	2 (13)	12 (19)

Data are n (%) or median (IQR). Due to rounding, not all variables might add up to 100%

*ALL* acute lymphoid leukemia, *AML* acute myeloid leukemia,* CLL* chronic lymphocytic leukemia, *CML* chronic myeloid leukemia, *COVID-19*coronavirus disease 2019, *HL* Hodgkin lymphoma.* IQR* interquartile range,*MDS* myelodysplastic syndrome, *MM* multiple myeloma,* NHL* non-Hodgkin lymphoma,* Ph-MPN* Philadelphia chromosome-negative myeloproliferative neoplasms

^*^Data are missing for 7 patients. ^†^Defined as having received anticancer therapy within 30 days prior to COVID-19 diagnosis; data are missing for 2 patients. ‡Includes intensive and standard dosing. ^§^Single-agent hydroxyurea, chlorambucil, or cyclophosphamide. ^¶^Includes tyrosine kinase inhibitors, Bruton's tyrosine kinase inhibitors, Aurora kinase inhibitors, PI3K inhibitors, proteasome inhibitors and histone deacetylase inhibitors. ^‡‡^ Single-agent rituximab, obinutuzumab, nivolumab, durvalumab, or brentuximab vedotin. Daratumumab includes both monotherapy and combination therapy*.* ^ǁ^Includes lenalidomide, pomalidomide and thalidomide. **Includes azacitidine and decitabine. ^††^Includes transfusion and hematopoietic growth factor support

### Outcomes

Participant vital status (death from any cause vs alive) was the main study outcome. Study centers could update patient status through data cutoff (May 25, 2020). Observation time for individual patients was calculated from the date of SARS-CoV-2 positivity to the date of death or last information update.

### Statistical analysis

Potential pre-infection prognostic factors for mortality were analyzed in three steps: unadjusted, partially adjusted and adjusted analyses. The associations between mortality and therapies received for COVID-19 were determined according to clinical severity (dichotomized as mild/moderate or severe/critical). Hazard ratios (HRs) and 95% confidence intervals (CIs) were estimated with Cox regression analyses. Variables included in the models were pre-specified and restricted in number to limit model overfitting. Analyses were generated using SAS/STAT software, Version 9.4, SAS Institute Inc.

## Results

**Fig. 1:**
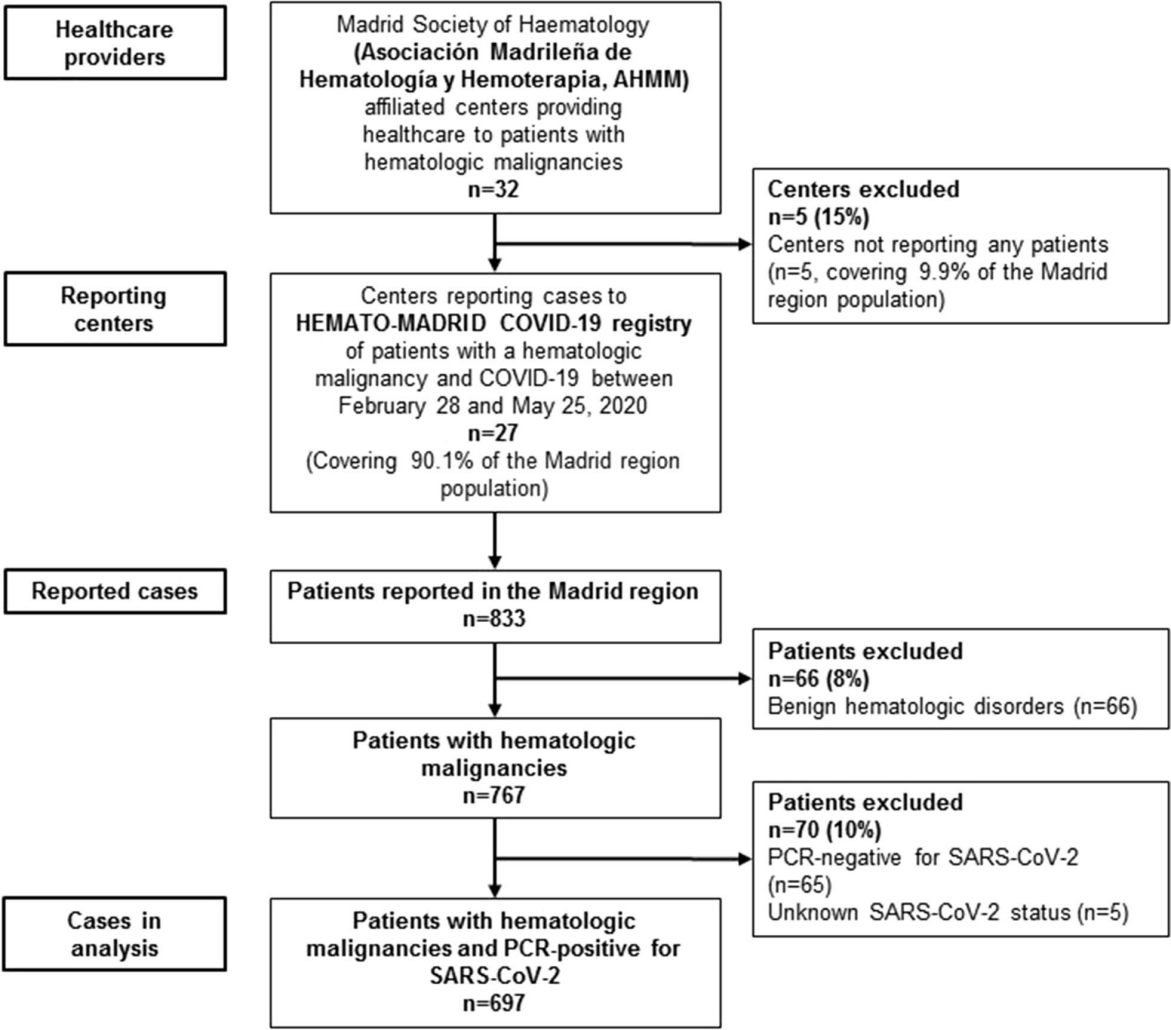
Patients with hematologic malignancies who were reported as having COVID-19 and who were included in the present analysis. Reporting hospitals included 22 of the 26 SERMAS regional health service centers (8/8 designated high complexity level hospitals; 10/12 intermediate complexity level; 4/6 low complexity level), and 5 of the 6 private centers

Of 833 patients reported to the HEMATO-MADRID COVID-19 registry by 27/32 healthcare providers, 697 were included in the present analyses (Fig. 1). The earliest PCR confirmation date in registered patients was February 28, 2020; during the 12-week reporting period, 5% of cases were reported in the 2 weeks prior to the start of lockdown, 75% in the following 4 weeks and the remaining 20% in the last 6 weeks.

Of the 697 patients, 479 (69%) had a lymphoid malignancy, including 187 (27%) with non-Hodgkin lymphoma and 137 (20%) with multiple myeloma, and 218 (31%) had a myeloid malignancy, including 78 (11%) with myelodysplastic syndrome (MDS), 61 (9%) with acute myeloid leukemia (AML) and 63 (9%) with Philadelphia chromosome (Ph)-negative myeloproliferative neoplasms (Table 1). Overall median age of patients was 72 years (IQR 60–79), and 413/690 (60%) were male. Comorbidities were present in 80% of patients, the commonest being hypertension (40%), cardiac disease (20%) and diabetes (17%) (Table 1).

In total, 405 (59%) patients were receiving active antineoplastic treatment, including at least 75% of patients with multiple myeloma, AML, chronic myeloid leukemia (CML), and Philadelphia chromosome (Ph)-negative myeloproliferative neoplasms (MPNs). The rate was 31% in patients with chronic lymphocytic leukemia or myelodysplastic syndrome (Table 1). Overall, 127 (18%) patients were receiving conventional chemotherapy, 81 (12%) molecular targeted therapies, 45 (6%) immunomodulatory drugs and 44 (6%) monoclonal antibodies (26 (59%) were on single-agent anti-CD20 therapy, 13 (29%) daratumumab and 10% others). Among 79 (12%) patients with history of hematopoietic stem cell transplantation (HSCT), 52 had received an autologous and 27 an allogeneic transplant; median age was 61 years (IQR 53–63), and median time since transplantation was 22 months (IQR 8–56).

COVID-19 clinical severity was classified as severe/critical in 62% of patients, moderate in 23% and mild in 15% (Table 2); 46% of transplant recipients had severe/critical disease. Patients with severe/critical disease were older and more likely (51%/22%) to have ≥ 3 comorbidities than those with mild COVID-19 (9%) (Table 2). Overall, 87% of patients required hospitalization, and 13% received ambulatory management (Table 1); 55 (8%) were admitted to an ICU (Table 2), most of whom had organ function damage, including 40/55 (73%) with acute respiratory distress syndrome and 2 (4%) with sepsis. Median age of ICU-admitted patients was 63 years (IQR 56–70).

**Table 2:** Characteristics and outcomes of patients with hematologic malignancies and COVID-19, according to COVID-19 clinical severity

	Clinical severity of COVID-19			
	Mild	Moderate	Severe	Critical
	n (%) shown as proportions of patients within a row/category			
Total (n = 692)*	104 (15)	159 (23)	290 (42)	139 (20)
Age, years^†^	63 (50–73)	71 (58–79)	74 (66–81)	72 (63–81)
18–49 (n = 72)	26 (36)	17 (24)	20 (28)	9 (13)
50–59 (n = 93)	23 (25)	27 (29)	28 (30)	15 (16)
60–69 (n = 130)	19 (15)	27 (21)	51 (39)	33 (25)
70–79 (n = 225)	23 (10)	50 (22)	110 (49)	42 (19)
> 80 (n = 171)	13 (8)	38 (22)	80 (47)	40 (23)
Sex^‡^				
Female (n = 276)	41 (15)	73 (26)	111 (40)	51 (18)
Male (n = 409)	62 (15)	83 (20)	178 (44)	86 (21)
Comorbidities^§^, n				
0 (n = 133)	40 (30)	32 (24)	38 (29)	23 (17)
1 (n = 180)	24 (13)	49 (27)	69 (38)	38 (21)
2 (n = 152)	17 (11)	33 (22)	71 (47)	31 (20)
≥ 3 (n = 192)	17 (9)	34 (18)	98 (51)	43 (22)
Hematologic malignancy				
Lymphoid malignancies (n = 477)^¶^	*75 (16)*	*115 (24)*	*196 (41)*	*91 (19)*
Non-Hodgkin lymphoma (n = 187)	33 (18)	53 (28)	71 (38)	30 (16)
Multiple myeloma (n = 136)^ǁ^	23 (17)	32 (23)	61 (45)	20 (15)
Chronic lymphocytic leukemia (n = 109)	9 (8)	21 (19)	47 (43)	32 (29)
Hodgkin lymphoma (n = 32)^ǁ^	6 (19)	7 (22)	13 (41)	6 (19)
Acute lymphoid leukemia (n = 13)	4 (31)	2 (15)	4 (31)	3 (23)
Myeloid malignancies (acute/subacute) (n = 139)	*17 (12)*	*24 (17)*	*62 (45)*	*36 (26)*
Acute myeloid leukemia (n = 61)	8 (13)	8 (13)	29 (48)	16 (26)
Myelodysplastic syndrome (n = 78)	9 (12)	16 (21)	33 (42)	20 (25)
Myeloproliferative malignancies (n = 76)**	*12 (16)*	*20 (26)*	*32 (42)*	*12 (16)*
Chronic myeloid leukemia (n = 16)	4 (25)	8 (50)	3 (19)	1 (6)
Ph-negative myeloproliferative neoplasms (n = 60)**	8 (13)	12 (20)	29 (48)	11 (18)
Hematopoietic stem cell transplantation^††^				
Autologous (n = 51)	14 (27)	15 (29)	16 (31)	6 (12)
Allogeneic (n = 27)	9 (33)	4 (15)	6 (22)	8 (30)
No (n = 585)	79 (14)	134 (23)	256 (44)	116 (20)
Active antineoplastic treatment (n = 691)^†,‡‡^				
Conventional chemotherapy (n = 127)	22 (17)	25 (20)	54 (43)	26 (20)
Low-intensity chemotherapy (n = 42)	8 (19)	10 (24)	15 (36)	9 (21)
Molecular targeted therapies (n = 81)^ǁ^	13 (16)	20 (25)	31 (38)	17 (21)
Monoclonal antibodies (n = 44)	6 (14)	10 (23)	22 (50)	6 (14)
Immunomodulatory drugs (n = 44)^ǁ^	5 (11)	8 (18)	26 (59)	5 (11)
Hypomethylating agents (n = 33)	1 (3)	10 (30)	17 (52)	5 (15)
Supportive care (n = 12)	0	4 (33)	7 (58)	1 (9)
Not detailed (n = 22)^¶^	1 (5)	6 (27)	10 (45)	5 (23)
No active therapy (n = 286)	47 (16)	66 (23)	108 (38)	65 (23)
Care setting				
Ambulatory care (n = 89)	57 (64)	18 (20)	13 (15)	1 (1)
Hospital care (n = 603)*	47 (8)	141 (23)	277 (46)	138 (23)
Intensive care unit (n = 55)^§§^	2 (4)	1 (2)	10 (18)	42 (76)
Outcome				
Survivors (n = 465)^¶^	101 (22)	147 (32)	170 (36)	47 (10)
Non-survivors (n = 227)**	3 (1)	12 (5)	120 (53)	92 (41)

COVID-19 severity classification followed WHO guidelines (20). Severe disease was defined as: bilateral lung infiltrates on chest imaging that were not fully explained by congestive heart failure or other forms of volume overload; tachypnea (≥ 30 breaths/min); oxygen saturation ≤ 90% at rest; and/or PaO_2_/FIO_2_ ratio < 300 mmHg. ‘Critical’ severity was defined as patients presenting with sepsis/septic shock, acute respiratory distress syndrome or multiple organ dysfunction/failure

Data are n (%) or median (IQR). Due to rounding, not all variables might add up to 100%

*COVID-19* coronavirus disease 2019, *IQR* interquartile range, *Ph-negative* Philadelphia chromosome-negative

Data are missing for *5 patients, ^†^6 patients, ^‡^12 patients, ^§^40 patients, ^¶^2 patients, ^ǁ^1 patient, **3 patients, ^††^31 patients, ^§§^12 patients

^‡‡^Defined as having received anticancer therapy within 30 days prior to COVID-19 diagnosis

Of 697 patients, 230 (33%) died, of whom 221 (96%) were hospitalized; 28/55 (51%) ICU-admitted patients died. Death was attributable to COVID-19 in 202 (88%) patients. Median time from confirmation of COVID-19 to death was 9 days (IQR 5–18). Median follow-up time for survivors was 43 days (IQR 32–53). Clinically relevant variables associated with increased mortality after multivariable adjustment (Table 3) were increasing age > 60 years, > 2 comorbidities (HR 1.4, 95% CI 1.05–1.90, vs ≤ 2 comorbidities), AML (vs non-Hodgkin lymphoma), and active antineoplastic treatment with monoclonal antibodies vs no active therapy; there was 50% increased mortality in patients receiving conventional chemotherapy vs no active therapy (HR 1.50, 0.99–2.29, *p* value 0.0561). Prognostic variables associated with lower mortality included Ph-negative MPNs (HR 0.33 vs non-Hodgkin lymphoma) and active treatment with hypomethylating agents (HR 0.47 vs no active treatment). Mortality rate among patients who underwent transplantation was 18%.

**Table 3:** Prognostic factors for mortality in patients with hematologic malignancies and COVID-19 (n = 697): time-to-event analysis

Pre-infection prognostic factors	Unadjusted (univariable)	Partially adjusted* (partial multivariable)	Adjusted analysis (full multivariable model)^†^	
	HR^‡^ (95%CI)	HR^‡^ (95%CI)	HR^‡^ (95%CI)	*P* value
Age, years				
18–49	1 (ref)	1 (ref)	1 (ref)	–
50–59	2.18 (0.85–5.56)	1.60 (0.61–4.23)	1.79 (0.66–4.89)	0.25
60–69	3.40 (1.43–8.12)	2.73 (1.12–6.66)	3.17 (1.25–8.00)	0.015
70–79	5.09 (2.22–11.7)	4.17 (1.78–9.79)	5.20 (2.12–12.8)	< 0.001
≥ 80	9.29 (4.07–21.2)	7.37 (3.12–17.4)	10.1 (4.03–25.4)	< 0.001
Sex				
Female	1 (ref)	1 (ref)	1 (ref)	
Male	1.05 (0.81–1.37)	1.01 (0.77–1.33)	1.13 (0.85–1.51)	0.4
Comorbidities, n				
0	1 (ref)	1 (ref)	1 (ref)	
1	1.30 (0.82–2.06)	0.97 (0.60–1.56)	0.99 (0.60–1.64)	0.9
2	1.95 (1.25–3.04)	1.15 (0.72–1.84)	1.17 (0.72–1.92)	0.5
≥ 3	2.55 (1.67–3.88)	1.40 (0.89–2.20)	1.51 (0.95–2.40)	0.08
Hematologic malignancy				
Non-Hodgkin lymphoma	1 (ref)	1 (ref)	1 (ref)	
Multiple myeloma	1.08 (0.74–1.59)	0.86 (0.58–1.27)	0.80 (0.49–1.28)	0.4
Chronic lymphocytic leukemia	1.01 (0.74–1.65)	0.80 (0.52–1.24)	0.92 (0.56–1.51)	0.8
Hodgkin lymphoma	0.79 (0.39–1.60)	0.96 (0.47–1.96)	1.20 (0.56–2.58)	0.6
Acute lymphoid leukemia	0.43 (0.10–1.74)	1.02 (0.24–4.38)	1.52 (0.36–6.58)	0.6
Acute myeloid leukemia	1.39 (0.88–2.19)	1.47 (0.92–2.35)	2.22 (1.31–3.74)	0.003
Myelodysplastic syndrome	1.43 (0.93–2.19)	0.86 (0.52–1.24)	1.14 (0.68–1.90)	0.6
Chronic myeloid leukemia	0.32 (0.08–1.32)	0.33 (0.08–1.36)	0.37 (0.08–1.70)	0.20
Ph-negative myeloproliferative neoplasms	0.52 (0.28–0.97)	0.33 (0.17–0.64)	0.33 (0.14–0.81)	0.015
Hematopoietic stem cell transplantation				
No	1 (ref)	1 (ref)	1 (ref)	
Autologous	0.45 (0.23–0.87)	0.90 (0.36–2.26)	0.79 (0.31–2.07)	0.6
Allogeneic	0.46 (0.19–1.11)	0.57 (0.28–1.19)	0.56 (0.27–1.19)	0.13
Active antineoplastic treatment^§^				
No active therapy	1 (ref)	1 (ref)	1 (ref)	
Conventional chemotherapy	1.04 (0.73–1.48)	1.56 (1.08–2.27)	1.50 (0.99–2.29)	0.0561
Low-intensity chemotherapy	0.50 (0.24–1.03)	0.46 (0.22–0.95)	1.04 (0.43–2.50)	0.9
Molecular targeted therapy	0.74 (0.47–1.18)	0.74 (0.45–1.22)	1.07 (0.61–1.88)	0.8
Monoclonal antibodies	1.37 (0.83–2.28)	1.73 (1.02–2.93)	2.02 (1.14–3.60)	0.016
Immunomodulatory drugs	1.08 (0.64–1.84)	1.14 (0.66–1.98)	1.64 (0.82–3.24)	0.16
Hypomethylating agents	1.03 (0.55–1.92)	0.74 (0.45–1.22)	0.47 (0.23–0.94)	0.032
Supportive care	2.01 (0.93–4.33)	1.15 (0.53–2.50)	1.50 (0.66–3.40)	0.3
Not detailed	0.88 (0.43–1.82)	0.86 (0.40–1.86)	0.86 (0.12–6.40)	0.9

*CI* confidence interval, *COVID-19* coronavirus disease 2019, *Ph-negative* Philadelphia chromosome-negative, *Ref* reference group

*Partial adjustment: multivariable analysis, all factors were adjusted by age, sex and comorbidity count. ^†^Full model: multivariable analysis, all variables were included in the model—including age, sex, comorbidity count, type of hematologic malignancy and therapy. ^‡^HRs and 95% CI were estimated with Cox regression analyses. ^§^Defined as having received anticancer therapy within 30 days prior to COVID-19 diagnosis

Overall, 574 (82%) patients received antiviral therapy (with β-interferon in 50 patients as an immunity booster), the most common being hydroxychloroquine in combination with antiretrovirals, azithromycin, or both (Table 4). Additionally, 346 (50%), 318 (46%) and 132 (19%) patients received empirical antibiotics, systemic corticosteroids (mainly methylprednisolone and prednisone) and off-label tocilizumab, respectively. Patients with severe/critical COVID-19 who did not receive antiviral therapy had a higher risk of death than patients receiving any antiviral combination therapy (HR 2.20, 95% CI 1.44–3.35) on multivariable analysis (Table 4). Mortality in patients treated with tocilizumab differed according to clinical severity of COVID-19 (test for strata homogeneity, *p* < 0.0001), with a higher risk in patients with mild/moderate COVID-19 treated vs not treated with tocilizumab (HR 5.94).

**Table 4:** COVID-19 pharmacological therapies and association with mortality in patients with hematologic malignancies and COVID-19, according to clinical severity of COVID-19: time-to-event analysis

	N (%), patients	n, events	Clinical severity of COVID-19					
			Mild/moderate			Severe/critical		
Patients with data on clinical severity of COVID-19	692 (100)	227	263 patients; 15 events			429 patients; 212 events		
Analysis of association with mortality			Univariable	Multivariable*		Univariable	Multivariable^†^	
			HR^‡^ (95% CI)		*P* value	HR^‡^ (95% CI)		*P* value
COVID-19 pharmacological therapy								
Antiviral therapy								
Hydroxychloroquine	558 (81)	184	0.92 (0.29–2.92)	1.22 (0.33–4.56)	0.8	0.40 (0.28–0.57)	0.38 (0.27–0.56)	< 0.001
Azithromycin	276 (40)	90	1.25 (0.44–3.52)	1.57 (0.54–4.56)	0.6	0.69 (0.52–0.90)	0.67 (0.58–0.89)	0.006
Antiretrovirals	337 (49)	116	1.16 (0.42–3.22)	1.70 (0.57–5.04)	0.4	0.77 (0.59–1.01)	0.91 (0.69–1.20)	0.5
β-interferon	50 (7)	27	1.28 (0.17–9.78)	2.12 (0.27–16.8)	0.5	1.53 (1.01–2.32)	1.52 (1.00–2.30)	0.051
Antiviral combination therapy								
No therapy	116 (17)	33	1 (Ref)	1 (Ref)		1 (Ref)	1 (Ref)	
Hydroxychloroquine alone	86 (12)	34	1.11 (0.19–6.69)	1.19 (0.16–8.60)	0.8	0.72 (0.43–1.20)	0.54 (0.31–0.92)	0.024
Hydroxychloroquine and azithromycin	148 (21)	40	0.66 (0.11–3.93)	0.94 (0.13–6.71)	0.6	0.35 (0.22–0.58)	0.34 (0.21–0.57)	< 0.001
Hydroxychloroquine and antiretrovirals	208 (30)	68	1.02 (0.23–4.58)	1.52 (0.27–8.63)	0.9	0.43 (0.28–0.68)	0.48 (0.30–0.77)	0.002
Hydroxychloroquine, azithromycin and antiretrovirals	116 (17)	42	1.80 (0.36–8.96)	4.57 (0.70–29.7)	0.14	0.40 (0.25–0.66)	0.40 (0.24–0.67)	< 0.001
Adjuvant therapy								
Systemic corticosteroids	318 (46)	133	2.43 (0.86–6.82)	2.34 (0.80–6.76)	0.13	0.88 (0.67–1.16)	1.01 (0.76–1.35)	0.9
Tocilizumab	132 (19)	51	5.07 (1.61–15.9)	5.94 (1.80–19.6)	0.002	0.62 (0.45–0.86)	0.87 (0.62–1.23)	0.4

*CI* confidence interval, *COVID-19* coronavirus disease 2019, *HR* hazard ratio

^*^Multivariable analyses for the mild/moderate severity group are adjusted for age only, due to the limited number of events. ^†^Multivariable analyses for the severe/critical severity group are adjusted for age (years), sex and comorbidity count, except for the analysis of β-interferon, which was adjusted for age only. ^‡^HRs and 95% CI were estimated with Cox regression analyses

## Discussion

To our knowledge, this is the first large-scale case series describing the epidemiology and outcome of COVID-19 in patients with hematologic malignancies. To date, only small case series in this setting have been reported [14, 15, 21, 22] in mainly hospitalized patients, whereas our study included both inpatients and outpatients. Our findings show that patients with hematologic malignancies and COVID-19 have threefold–fourfold higher rates of severe/critical disease (62% vs 15%) and mortality (33% vs 10%) compared to COVID-19 cases in the general population [23–25]. Clinical severity of COVID-19 was worse, and mortality rates were higher among older patients and those with a greater number of comorbidities and varied by type of hematologic malignancy and active antineoplastic treatment. Rates of severe/critical COVID-19 and mortality in our study were higher than reported in patients with solid tumors (26–43% and 13–28%; respectively) [9].

Despite the high societal impact of COVID-19 in Spain, the ENE-COVID nation-wide, population-based study reported a SARS-CoV-2 seroprevalence of 11.5% for the Madrid region [26], which is clearly insufficient to provide herd immunity. Together with our study, these findings have important policy implications, including the need for increased surveillance for SARS-CoV-2 in patients with hematologic malignancies.

Among the strengths of this study are the prospective and comprehensive collection of clinical and outcome data, and the use of multivariable analysis to identify independent risk factors for death. Our patient series is highly representative of this population as, in Spain, health care for all patients with hematologic malignancies is centralized in hospitals. Furthermore, all hospitals in the Madrid region were under the governance of the Madrid Health System authorities and were following the guidelines of the Spanish Health Minister. We therefore believe that the mortality rate in our study reflects the true mortality rate in patients with hematologic malignancies and COVID-19 that contacted the healthcare system during the growth phase of the pandemic. Another strength was the selection of a restricted number of prognostic factors based on clinical features for determining associations with mortality rate. These factors could be utilized in a prognostic model to stratify patients with hematologic malignancies and to implement preventive strategies for future healthcare crises.

Key risk factors for clinical severity and mortality previously reported in the general population (e.g., older age, higher number of comorbidities) were validated in our study. Notably, the median age of patients in our series was higher than in the general population with COVID-19 (72 vs 60 years), with 32% of cases occurring in patients aged 70–79 years; in the general population, COVID-19 cases were more uniformly distributed across age groups [23]. Additionally, our findings highlight that the type of hematologic malignancy was associated with COVID-19 mortality. Our study showed relatively higher mortality rates in patients with AML (44%) and myelodysplastic syndrome (42%) and relatively lower rates in patients with Ph-negative MPNs (19%) and CML (13%), consistent with a Chinese study of 5 patients with CML receiving tyrosine kinase inhibitor (TKI) therapy [27].

The differential outcomes from COVID-19 between patients with different hematologic malignancies could be associated with multiple factors. On multivariate analysis, we found that type of active antineoplastic treatment appeared associated with mortality from COVID-19. Patients receiving monoclonal antibody-based therapy had a significantly greater (HR 2.02) risk of death vs those not receiving active antineoplastic treatment, while those receiving active conventional chemotherapy were 50% more likely to die from COVID-19. By contrast, there was a significant 53% lower mortality among patients receiving hypomethylating agents (HMA). Among the 33 patients treated with HMA, 45% were MDS and 52% were AML; 23% of patients with MDS or AML were treated with HMAs.

The association between low-intensity chemotherapy and lower COVID-19 mortality in our partially adjusted analysis could be in part because nearly half of patients with Ph-negative MPNs were receiving this chemotherapy. Interestingly, acknowledging that the number of patients with CML was low, 88% were receiving molecular targeted therapies (TKI therapy), and mortality rate was only 13%. Kinase inhibitor-targeted therapy was demonstrated to block dissemination of SARS-CoV and Middle East respiratory syndrome coronavirus and is being investigated as a potential therapeutic approach against SARS-CoV-2 [28]. Our findings suggest that patients with CML may even garner some protection against poor outcomes with COVID-19 due to their TKI therapy; discontinuing such therapy out of fear of COVID-19 might not be warranted.

The clinical characteristics, management and outcome of COVID-19 in patients undergoing HSCT remain unknown; guidelines are being generated by various organizations [29, 30]. Our real-world data showed a mortality rate of 18%. However, this figure should be interpreted with caution as both type of transplant and time from transplant (IQR 8–56 months) were heterogeneous. Our data suggest that life-saving transplantation should not be delayed in patients with hematologic malignancies, although close monitoring is of paramount importance.

Although the majority of the 230 deaths in our case series occurred during hospitalization, only 55 (8%) patients were admitted to an ICU. This rate is consistent with reports from the UK [9] and USA [31], in which the incidence of ICU admission among patients with all types of cancer and COVID-19 was 6–14%. We were not able to determine from our dataset whether a diagnosis of a hematologic malignancy decreases a patient’s chances of accessing such intensive support, for example due to equipment and/or personnel shortages. In our cohort, overall mortality rate in patients admitted to an ICU was 51%, suggesting that many patients with hematologic malignancy can survive COVID-19 and require equivalent access to ICU care.

There are currently no approved treatment options for patients with COVID-19 in Europe, and no clear recommendations can be made regarding specific therapies due to limited data and unknown risk: benefit profiles. Even fewer such data are available for patients with hematologic malignancies. Our study is the first in which the effect of COVID-19 treatments has been studied in such patients with different degrees of clinical severity of COVID-19. Interestingly, our findings showed that in patients with severe/critical COVID-19, not receiving any antiviral therapy was associated with higher mortality than being treated with any antiviral combination therapy. However, this was a non-randomized study and residual confounding by indication may explain this finding. These data provide a rationale for including patients with hematologic malignancies in investigational strategies of antiviral therapy. Regarding the effect of corticoids, although some clinical trials have showed that corticoids have a survival benefit, in patients with hematological malignancies, we have not seen this effect. Notably, we also observed that the mortality rate in patients with mild/moderate COVID-19 was nearly sixfold higher among those treated with, vs not receiving, tocilizumab. Further, tocilizumab was not associated with any benefit in patients with severe/critical COVID-19. As no reference guidelines were available, these results may be related to the expected high variability in the criteria used for prescribing tocilizumab, doses administered and clinical severity of the disease at the time of drug administration. These findings call for optimizing precision medicine strategies when designing controlled studies on the use of tocilizumab.

### Limitations

Our study has some limitations. First, this was primarily an observational cohort study designed for rapid patient accrual during the nonlinear growth phase of the COVID-19 outbreak. For this reason, our study introduces uncertainty into the exact timing of therapeutic intervals, as required to meet IRB regulatory requirements. Second, although to the best of our knowledge we included all patients with hematologic malignancies and COVID-19, the true number of such patients might have been higher because of the low rate of testing or misdiagnoses at the beginning of the pandemic and including only patients who contacted the healthcare system. More data on the prevalence and outcomes of COVID-19 in asymptomatic patients with hematologic malignancies could emerge as hospital systems implement more comprehensive testing of all patients seeking care. Third, data on clinical symptoms, laboratory findings, disease status and details on therapy were missing. We did not collect data on contacts although during the first wave of the COVID-19 pandemic in Madrid, we expected most patients to be infected by community transmission. Finally, our case series incorporates a heterogeneous patient population with multiple different hematologic malignancies; further studies in patients with specific malignancies [32] are needed, as it is possible that type of malignancy and disease status may affect the clinical course of COVID-19. Nevertheless, our study included a large number of patients with some malignancies, which could overcome in part this perceived limitation.

## Conclusions

In conclusion, our findings support the vulnerability of patients with hematologic malignancies in the COVID-19 pandemic and provide several important considerations for clinical care. In addition to the previously determined risk factors for older age and multiple comorbidities, patients with AML and those currently receiving or who have recently received antineoplastic therapy with monoclonal antibodies are at increased risk of death; those receiving conventional/intensive cytotoxic chemotherapy may be at higher risk; further studies should identify which conventional chemotherapies are associated with increased mortality. By contrast, we found evidence that active treatment with hypomethylating agents may be associated with more favorable outcomes. The higher mortality in patients with hematologic malignancies and severe/critical COVID-19 who did not receive antiviral therapy provides the rationale for including these patients in investigational strategies. Further studies and long-term follow-up are required to validate these criteria for risk-stratifying patients with hematologic malignancies in a future healthcare crisis and for defining appropriate timing and types of antineoplastic treatments.

## Data Availability

The datasets used and/or analyzed during the current study are available from the corresponding author on a reasonable request.

## Abbreviations

SARS-CoV-2: Severe acute respiratory syndrome coronavirus 2
COVID-19: Coronavirus disease 2019
WHO: World Health Organization
ICU: Intensive care unit
AMHH: Madrid Society of Hematology—Asociación Madrileña de Hematología y Hemoterapia
SERMAS: Madrid Regional Health Service—Servicio Madrileño de Salud
IRB: Institutional Review Board
IQR: Interquartile range
HR: Hazard ratio
CI: Confidence interval
AML: Acute myeloid leukemia
CLL: Chronic lymphocytic leukemia
Ph: Philadelphia chromosome
CML: Chronic myeloid leukemia
MPN: Myeloproliferative neoplasms
HSCT: Hematopoietic stem cell transplantation
